# SNAREs-SAP: SNARE Proteins Identification With PSSM Profiles

**DOI:** 10.3389/fgene.2021.809001

**Published:** 2021-12-20

**Authors:** Zixiao Zhang, Yue Gong, Bo Gao, Hongfei Li, Wentao Gao, Yuming Zhao, Benzhi Dong

**Affiliations:** ^1^ College of Information and Computer Engineering, Northeast Forestry University, Harbin, China; ^2^ Department of Radiology, The Second Affiliated Hospital, Harbin Medical University, Harbin, China

**Keywords:** SNARE proteins, position-specific scoring matrix, machine learning, support vector machine, SVM-RFE-CBR

## Abstract

Soluble N-ethylmaleimide sensitive factor activating protein receptor (SNARE) proteins are a large family of transmembrane proteins located in organelles and vesicles. The important roles of SNARE proteins include initiating the vesicle fusion process and activating and fusing proteins as they undergo exocytosis activity, and SNARE proteins are also vital for the transport regulation of membrane proteins and non-regulatory vesicles. Therefore, there is great significance in establishing a method to efficiently identify SNARE proteins. However, the identification accuracy of the existing methods such as SNARE CNN is not satisfied. In our study, we developed a method based on a support vector machine (SVM) that can effectively recognize SNARE proteins. We used the position-specific scoring matrix (PSSM) method to extract features of SNARE protein sequences, used the support vector machine recursive elimination correlation bias reduction (SVM-RFE-CBR) algorithm to rank the importance of features, and then screened out the optimal subset of feature data based on the sorted results. We input the feature data into the model when building the model, used 10-fold crossing validation for training, and tested model performance by using an independent dataset. In independent tests, the ability of our method to identify SNARE proteins achieved a sensitivity of 68%, specificity of 94%, accuracy of 92%, area under the curve (AUC) of 84%, and Matthew’s correlation coefficient (MCC) of 0.48. The results of the experiment show that the common evaluation indicators of our method are excellent, indicating that our method performs better than other existing classification methods in identifying SNARE proteins.

## 1 Introduction

N-ethylmaleimide sensitive factor (NSF) (Whiteheart et al., 2001) protein and soluble NSF attachment proteins (SNAPS) (Whiteheart et al., 1993) are two essential factors for protein transport between membranes (Hohl et al., 1998) (Hanson et al., 1997). They were first discovered as essential proteins for protein transport from donor to receptor subcellular structures during the processes of Golgi modification and secretion. The discovery of these two proteins led to the discovery of multiple receptor proteins on transport vesicles and plasma membranes and snap receptors, which are collectively called soluble N-ethylmaleimide-sensitive factor activating protein receptor (SNARE) proteins (Ungar and Hughson, 2003; Zhao et al., 2019). According to the SNARE theory, exocytosis and secretory processes are completed by precise coordination between SNARE proteins. The specificity of membrane fusion is based on the specific binding of SNARE protein members. At the molecular level, when the transport vesicle is close to the target membrane, syntaxin1A/B on the target membrane receives a signal to recognize, approach and combine with SNAP25, which is also located on the target membrane. At the same time, VAMP2 (q-snare) on the transport vesicle also recognizes (Kweon et al., 2003), draws close to and binds to form a 7S R-Q-SNARE complex, which guides the attachment and fusion of the transport vesicle and the target membrane, leading to the secretion of substances in the transport vesicle into the new subcellular structure or out of the cell through exocytosis, completing the intracellular transport and extracellular exocytosis and secretion processes.

The binding sites of SNARE proteins are specific, which is the reason for the specificity and precision of exocytosis and secretion in different organisms and organs (Fasshauer et al., 1998; Yin et al., 2021). SNARE theory convincingly explains the key role of synapses in the process of nerve impulse transmission at the molecular level (Chen and Scheller, 2001). Its new insights in the fields of molecular neurobiology and endocrinology have made research on SNARE proteins a hot spot in the basic life sciences worldwide. Such findings greatly enrich understanding of the regulation of intracellular information transmission, substance transport and exocytosis and secretion at the molecular level and improve knowledge of the interaction between proteins and the plasma membrane (Liu et al., 2019a; Wang et al., 2020a; Xu et al., 2021).

Due to the important roles of SNARE proteins in cell biology, research on SNARE proteins is also developing, and a variety of technologies are used to study SNARE proteins (Wang et al., 2020b; Yin et al., 2020), including the establishment of a SNARE protein database, the retrieval and classification of SNARE proteins, bioinformatics technology that was used to predict the role of SNARE proteins, and construction of a neural network model to recognize SNARE proteins.

With the development of computational biology, the application of machine learning to bioinformatics continues to be deep and widespread (Jiang et al., 2013; Tao et al., 2020; Zhao et al., 2021). Machine learning is complex and cross disciplinary across multiple fields (Cheng, 2020). Machine learning obtains new knowledge through learning from pre-existing knowledge and can continuously advance itself based on large quantities of this pre-existing knowledge and skills. Research on machine learning includes the study of computer algorithms, using data and previous techniques to improve the performance of computer algorithms. Machine learning also has significant implications for the development of artificial intelligence, through which computers continuously progress along a path of constant intelligence. A typical way to predict proteins is to transform each protein sequence into a numerical eigenvector used to represent the protein sequence, training a classification model based on the eigenvectors of the training samples and the labels. After feature construction, the classifiers that make predictions about proteins include covariant discriminant (CD) (Chou, 2000), support vector machine (SVM) (Hua and Sun, 2001), K-nearest neighbor (KNN) (Shen and Chou, 2006), deep learning and ensemble classifiers (Shen and Chou, 2006).

In this study, based on SVM classifier (Liu et al., 2010), we constructed a model to recognize SNARE proteins. We use position-specific scoring matrix (PSSM) profiles of protein sequences to extract features (Kumar et al., 2008), process the feature data by the min-max normalization method, build a model based on SVM, train the model with 10-fold cross validation and measure the performance of the model on an independent dataset.

## 2 Materials and Methods

**FIGURE 1:**
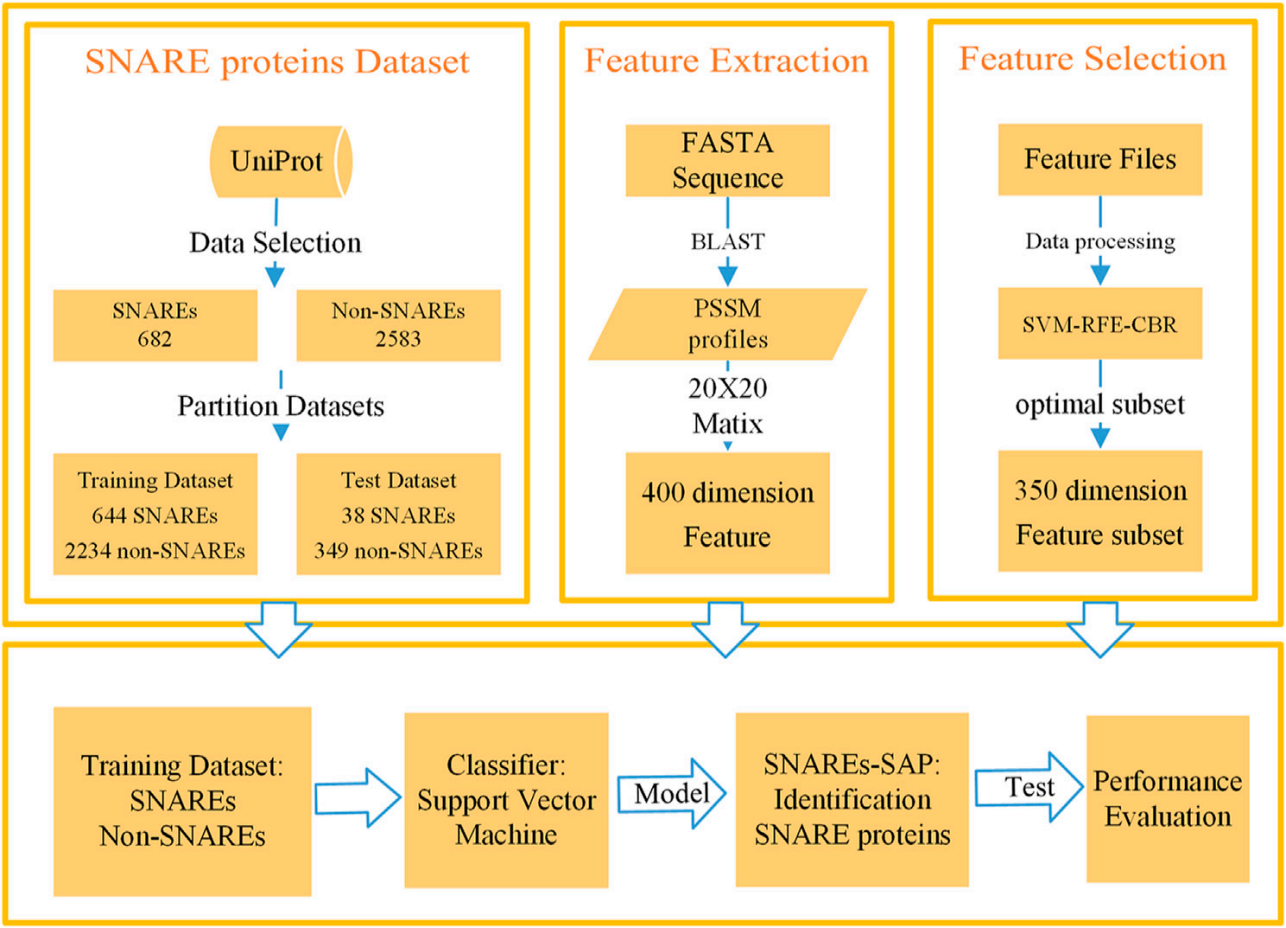
Flow chart of SNARE proteins recognition based on PSSM profiles matrix and SVM.

We developed a method to recognize SNARE proteins based on PSSM (Chou and Shen, 2007; Liu et al., 2019b; Hong et al., 2020a; Hong et al., 2020b) profiles and SVM. Method steps include data collection, data processing, feature extraction, feature selection, model training, and model performance evaluation. The overall flow of our designed method is summarized in Figure 1, and each section in the figure is described in detail in the following sections. We carried out experiments through the above process, constantly adjusted in our experiment, and finally constructed an excellent method to identify SNARE proteins. The following is a detailed description of the method.

### 2.1 Feature Extraction

It is very important to select good feature information for protein recognition (Zuo et al., 2017; Zheng et al., 2019; Tang et al., 2020a; Guo et al., 2020; Zhang et al., 2021). We chose the method based on PSSM profiles to extract the feature information of protein sequence data. We use the National Center for Biotechnology Information basic local alignment search tool (NCBI-BLAST) and select a non-redundant (NR) protein sequence database as a comparison dataset. We use the prepared SNARE protein FASTA sequence files to generate PSSM profiles. Each amino acid of the original sequence in the PSSM profiles consists of a vector of 20 values. Then, we transform the original PSSM files into PSSM profiles with 400 dimensions. Finally, 400-dimensional data are extracted as the feature data of each protein sequence for the experiment.

### 2.2 Data Processing

The feature data in the datasets are seriously unbalanced, especially the ratio of positive samples to negative samples in the independent dataset, which varies tremendously. The model would exhibit the problem of poor generalization, and the applicability would be low, so it is unable to effectively identify SNARE proteins. Therefore, we need to choose the appropriate method to deal with the data. In this study, the data processing methods we chose included Z-score standardization, min-max normalization and L2 regularization.

Normalization: Data can be changed to [0, one] ranges using the normalization method. Normalization, as an effective way to simplify calculation and scale down data values, can change the absolute values of data in the dataset into a relationship of some relative value. After normalization, the data can be calculated conveniently and quickly. This is mainly for the convenience of data processing, mapping the data to the range of 0–1, which will be convenient and fast to use. The method is defined as:

$$\begin{array}{c} {\text{x}}^{\text{*}}=\frac{\text{x}-\text{min}}{\text{max}-\text{min}},\# \end{array}$$ (1)

The distribution of original data can be changed by normalization, and then the weights of each feature dimension can be balanced by varying the feature dimension, such as converting the distribution of data from planar to circular. Normalization can remove the influence of dimensionality on the experimental results by reducing the difference in dimensionality. After normalization, the data of different variables can be compared. Although the maximum and minimum values of the resulting data in the normalization process are affected by outliers in the dataset, and the resulting data are less robust, normalization does improve the accuracy of iterations in the operational data process as well as the efficiency of data convergence.

### 2.3 Feature Selection

Feature selection refers to sorting features by suitable techniques and algorithms and filtering out the better characterized subset of features based on the sorted results; this is a common technique in bioinformatics (Cheng et al., 2018; Zhu et al., 2019; Zhao et al., 2020a; Zhao et al., 2020b; Shao and Liu, 2021; Yu et al., 2021). After feature selection, the optimal feature subset selected from existing features is used to build the model, which can improve the performance of the model. Feature selection is a very important part of building models for pattern recognition and is a high priority in data processing (Wei et al., 2018; Xue et al., 2018; Li et al., 2020a; Yang et al., 2020a; Su et al., 2020; Wei et al., 2020; Yu et al., 2020; Zhang et al., 2020; Zheng et al., 2020; Wang et al., 2021a; Shang et al., 2021; Shao et al., 2021). Selecting the effective features from the original feature dataset and removing the redundant features can reduce the dimensionality of the feature data, and using more effective feature data can improve the performance of the model. Our original feature is based on PSSM to extract 400 dimensional features. In these original feature spaces, there will be irrelevant, noisy, and redundant features. Suitable feature selection methods with excellent performance are required for accurate screening of redundant features. In our experiment, we finally chose the SVM-RFE-CBR (Yan and Zhang, 2015) algorithm to screen features after comparing multiple feature selection methods. The algorithm ranks the importance of features and selects the optimal subset of features based on the sorted results.

SVM-RFE-CBR is an improved algorithm based on support vector machine recursive feature elimination (SVM-RFE), which introduces the strategy of correlation deviation reduction (CBR) into the process of feature elimination. SVM-RFE estimates feature importance based on the coefficient of the SVM model, and it is a powerful feature selection algorithm. There are linear and nonlinear versions. The SVM-RFE-CBR method adds the correlation reduction strategy (CBR) to the SVM-RFE algorithm to reduce the potential deviation of the algorithm, and the result of feature selection is improved by the integrated CBR strategy. SVM-RFE uses the sequential backward selection algorithm in SVM, which is based on the principle of maximum interval. During the model training process, SVM-RFE sort features based on the score of every feature, deletes the feature with the lowest score, puts the remaining feature data into the next round of training of the model, and finally outputs the feature sort result to a table. The optimal feature subset can be selected according to the results of sorting. SVM is an excellent machine learning classification algorithm. The feature sort result derived from the SVM model has better performance, and it is also more convenient for subsequent experiments.

### 2.4 Support Vector Machine

SVM is currently a commonly used classifier in machine learning that classifies data by supervised learning (Cheng et al., 2019a; Cheng et al., 2019b). SVM is commonly used in data dichotomization. In addition, SVM can classify nonlinearly by using the kernel function (Ding et al., 2020a; Liu et al., 2020a; Yang et al., 2020b). SVM was developed from the generalized portrait algorithm in pattern recognition. The basic idea of SVM is to construct a model that separates the dataset accurately according to the geometric interval of the hyperplane with the maximum separation of samples. SVM can map the features of a dataset to points in space and draw a line to distinguish these points effectively. SVM uses a hinge loss function to computationally predict the presence of empirical risk, and a regularization term is added to ensure its robustness and correct rate. The process of SVM: Suppose the training set is $\left\{\left({\text{x}}_{\text{i}},{\text{y}}_{\text{i}}\right)\right\}\begin{array}{c} \text{N}\\ \text{i}=1 \end{array}$ , ${\text{x}}_{\text{i}}\in {\text{R}}^{\text{D}}$ , ${\text{y}}_{\text{i}}\in \left\{+1,\;-1\right\}$ , ${\text{x}}_{\text{i}}$ is the *i*th sample, N is the sample size, and D is the number of sample features. SVM finding the optimal classification hyperplane. ω⋅x+b=0 The optimization problems that SVM needs to solve are:

$$\begin{array}{l} \begin{array}{c} \text{min }\frac{1}{2}{\left|\left|\text{ω}\right|\right|}^{2}+C\sum_{\text{i}=1}^{\text{N}}\varepsilon_{\text{i}}.\# \end{array}\\ \text{s}.\text{t}.{\text{ y}}_{\text{i}}\left(\text{ω}\cdot {\text{x}}_{\text{i}}+\text{b}\right)\geq 1-{\text{ε}}_{\text{i}},\text{ i}=1,\;2,\;\cdots ,\text{ N}\\ {\text{ε}}_{\text{i}}\geq 0,\text{ i}=1,\;2,\;\cdots ,\text{ N} \end{array}$$ (2)

Transforming the original problem into the dual problem:

$$\begin{array}{c} \min\frac{1}{2}\sum_{\text{i}=1}^{\text{N}}\sum_{\text{j}=1}^{\text{N}}{\text{α}}_{\text{i}}{\text{α}}_{\text{j}}{\text{y}}_{\text{i}}{\text{y}}_{\text{j}}\left({\text{x}}_{\text{i}}\cdot {\text{x}}_{\text{j}}\right)-\sum_{\text{i}=1}^{\text{N}}\alpha_{\text{i}} \end{array}.$$ (3)

$$\begin{array}{c} s.t.\;\sum_{\text{i}=1}^{\text{N}}{\text{y}}_{\text{i}}{\text{α}}_{\text{i}}=0 \end{array}.\#$$ (4)

$$0\leq {\text{α}}_{\text{i}}\leq \text{C},\text{ i}=1,\;2,\;\cdots ,{\text{ N α}}_{\text{i}}\text{ is a Lagrangian}$$

Finally, the solution of ω is:

$$\begin{array}{c} \omega =\sum_{\text{i}=1}^{N}{\text{α}}_{\text{i}}{\text{y}}_{\text{i}}{\text{x}}_{\text{i}}.\# \end{array}$$ (5)

When we use SVM to solve nonlinear problems, we need to choose the appropriate kernel function (Yang et al., 2021a) (Ding et al., 2020b) and then map the data to the high-dimensional space to solve the linearly inseparable problem of the data in the original space.

In the experiment, the Python version of a library for support vector machine (LIBSVM) was selected to build an SVM model and identify SNARE proteins. The selection of different kernel functions using LIBSVM as well as the settings of kernel parameters are described as follows: The kernel function (Ding et al., 2020c) of SVM includes the linear kernel function (LKF), polynomial kernel function (PKF), radial basis function (RBF), and sigmoid kernel function (SKF). Formulas corresponding to four kernel functions are as follows:

Linear kernel function defined as:

$$\begin{array}{c} \text{K}\left({\text{x}}_{\text{i}},{\text{x}}_{\text{j}}\right)={\text{x}}_{\text{i}}^{\text{T}}{\text{x}}_{\text{j}}.\# \end{array}$$ (6)

Polynomial kernel function:

$$\begin{array}{c} \text{K}\left({\text{x}}_{\text{i}},{\text{x}}_{\text{j}}\right)={\left({\nu \text{x}}_{\text{i}}^{\text{T}}{\text{x}}_{\text{j}}+\text{r}\right)}^{\text{d}},\;\nu >0.\# \end{array}$$ (7)

Radial basis functions:

$$\begin{array}{c} \text{K}\left({\text{x}}_{\text{i}},{\text{x}}_{\text{j}}\right)=\exp\left(-\text{ν}{\left|\left|{\text{x}}_{\text{i}}-{\text{x}}_{\text{j}}\right|\right|}^{2}\right),\;\nu >0.\# \end{array}$$ (8)

Sigmoid kernel function:

$$\begin{array}{c} \text{K}\left({\text{x}}_{\text{i}},{\text{x}}_{\text{j}}\right)=\tanh\left({\text{νx}}_{\text{i}}^{\text{T}}+\text{r}\right).\# \end{array}$$ (9)

ν, r, and d in formulas are parameters of kernel function.

Parameters are different in different kernel functions. ν in the formula represents the parameter gamma in the kernel function, the default of which is 1/K (K is the number of classes), and g is used to set it in the LIBSVM.

r in the formula represents the parameter r in the kernel function, the default of which is 0, and r is used to set it in the LIBSVM. d in the formula represents the parameter d in the kernel function; it is used to set the highest number of times in the polynomial kernel function, and its default value is 3.

SVM is a very powerful model that allows the decision boundary to be very complex and performs well on both low-dimensional data and high-dimensional data. SVM has been widely used in bioinformatics, binding protein prediction, protein methylation site prediction and so on. We use the LIBSVM of Scikit-learn library integration in Python to train and build the model. In our experimental process, we optimize the parameters according to the results and finally build the model with the best performance.

## 3 Results and Discussion

### 3.1 Dataset

Our research is devoted to constructing a method to recognize SNARE proteins. To establish a model to effectively distinguish SNARE proteins and non-SNARE proteins, we collected a SNARE protein dataset and a non-SNARE protein dataset for our prediction model. The dataset we use has been used by Le, N.Q.K. and V.-N. Nguyen (Le and Nguyen, 2019) previously. The data come from the UniProt database, which is the most informative and resource-free protein database. We collect all SNARE proteins from the UniProt database according to the keyword SNARE. To avoid the homology of the SNARE protein sequence data that we collect, we use BLAST to address the redundancy of the SNARE protein sequence and eliminate the redundant sequence. Finally, 682 SNARE protein sequences are obtained as a positive sample dataset. At the same time, we select vesicular transport proteins as negative samples to establish a non-SNARE protein dataset. We divide the two datasets into a cross-validation dataset and an independent test dataset, and the size and details of the datasets are summarized in Table 1.

**TABLE 1:** Summary of SNARE protein and non-SNARE protein datasets.

Dataset	SNARE	Non-SANRE	Total
Original dataset	682	2,583	3,265
Train dataset	644	2,234	2,878
Test dataset	38	349	387

Table 1 shows that SNARE proteins and non-SNARE proteins correspond to two datasets: a training dataset and an independent test dataset, both of which include positive samples and negative samples. We use the cross-validation method to train the model with the training dataset, evaluate the performance of the model developed in this study, and optimize the model by adjusting the parameters according to the results of the training dataset. The independent test dataset is used to test and measure the predictive ability of the prediction model we developed.

### 3.2 Performance Measurements

Our research aims to establish a model to predict whether an amino acid sequence is a SNARE protein. Therefore, we need to use universally acknowledged evaluation indicators to measure the performance of the model. When training the model, we choose 10-fold cross validation as the training model after various considerations and take the average value of the crossing validation results as the result of model training. We optimize the parameters of SVM, select the best parameters to build the model, and evaluate the performance of the model through an independent test dataset to avoid systematic deviation in the process of cross validation. This study adopts some standard evaluation indicators that are widely used in bioinformatics research (Shen et al., 2019a; Shen et al., 2019b; Ao et al., 2020; Li et al., 2020b; Liu et al., 2020b; Tang et al., 2020b; Yin et al., 2020; Chen et al., 2021). The standard evaluation indicators include sensitivity (Sn), specificity (Sp), accuracy (Acc), area under the curve (AUC), Mathew’s correlation coefficient (MCC), and F-score (Zhai et al., 2020; Wang et al., 2021b; Yang et al., 2021b). The calculation formulas are as follows (TP means true positive values, FP means false positive values, TN means true negative values, FN means false negative values):

$$\begin{array}{c} \text{Sensitivity}=\frac{\text{TP}}{\text{TP}+\text{FN}},\;0\leq Sn\leq 1.\# \end{array}$$ (10)

$$\begin{array}{c} \text{Speccificity}=\frac{\text{TN}}{\text{TN}+\text{FP}},\;0\leq Sp\leq 1.\# \end{array}$$ (11)

$$\begin{array}{c} \text{Accurarcy}=\frac{\text{TN}+\text{TP}}{\text{TP}+\text{TN}+\text{FN}+\text{FP}},\;0\leq \text{Acc}\leq 1.\# \end{array}$$ (12)

$$\begin{array}{c} \text{MCC}=\frac{\text{TP*TN}-\text{FP*FN}}{\sqrt{\left(\text{TP}+\text{FN}\right)\left(\text{TN}+\text{FN}\right)\left(\text{TP}+\text{FP}\right)\left(\text{TN}+\text{FP}\right)}},\;0\leq \text{MCC}\leq 1.\# \end{array}$$ (13)

$$\begin{array}{c} \text{F}-\text{score}=\frac{2\text{*TP}}{2\text{TP}+\text{FN}+\text{FP}},\;0\leq \text{F}-\text{score}\leq 1.\# \end{array}$$ (14)

In machine learning research, receiver operating characteristic (ROC) curves are usually used to test the prediction performance of the model. AUC is a floating-point number from 0 to one of ROC. The AUC value can reflect the quality of the model. The greater the value, the better the performance of the model. ROC curves and AUCs are commonly used to compare the performance of different models as machine learning performance indicators, which is very reliable. MCC is often used to measure imbalanced data sets, which is one of the most important indicators to measure the performance of two kinds of classification in machine learning. We use Python’s processing library to process data.

### 3.3 Performance Comparison With Different Feature Dimensions

**FIGURE 2:**
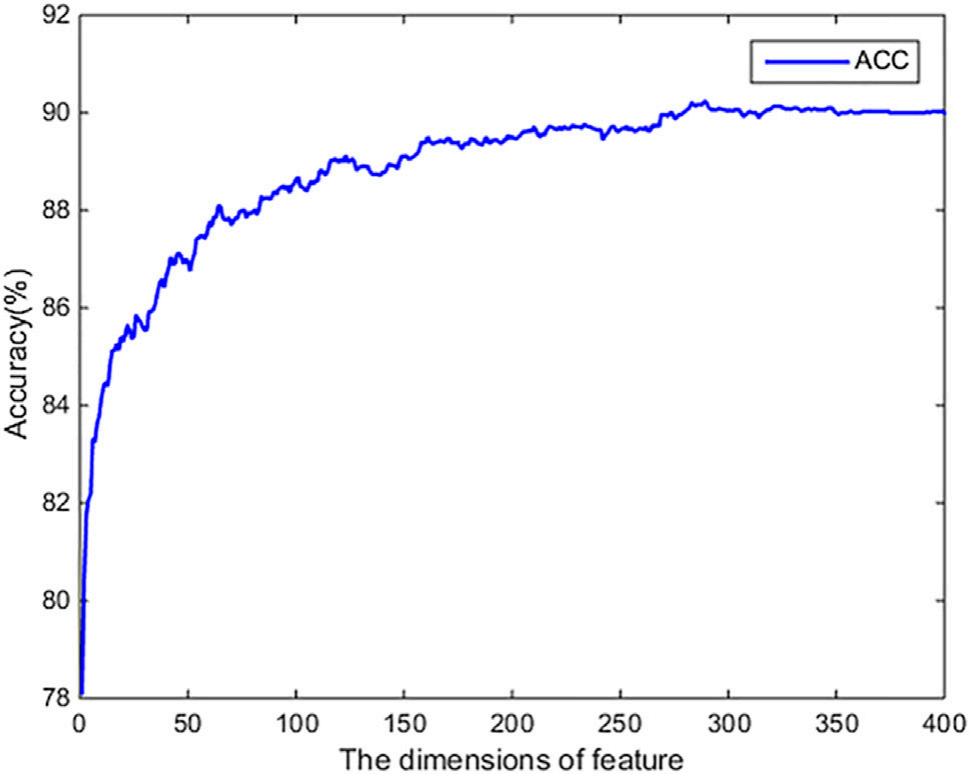
The results of dimension reduction by using SVM-RFE-CBR algorithm.

We use the SVM-RFE-CBR algorithm to evaluate the original 400-dimensional feature data. We use MATLAB to implement the SVM-REF-CBR algorithm to sort the features. When sorting features, a performance comparison will be given. The evaluation results are shown in Figure 2. From Figure 2, it can be found that the ACC achieved highest value, when the top 350-dimensional feature is used in the experiment. Therefore, we choose 350-dimensional feature data for the experiment.

We use the optimal 350-dimensional feature dataset after sorting for the experiment. First, 350-dimensional feature data are selected from the original feature training dataset and test dataset files according to the index obtained by the SVM-RFE-CBR algorithm. Then, the training dataset is 10-fold cross validated, and the model is optimized. After many experiments, the optimal parameters of SVM are obtained. When we choose the radial basis function, penalty coefficient (C) = “11”, gamma = “0.1”, the model achieves the optimal performance. At the same time, we also use the original 400-dimensional feature data for the experiment and choose the optimal parameterization in the experiment. The comparison of experimental results in different dimensions is shown in Table 2.

**TABLE 2:** Comparison of prediction results between SVM-RFE-CBR dimension reduction and original dimension.

Feature-dimension	Sn	Sp	Acc	AUC	MCC	F-score
350	**0.68**	**0.94**	**0.92**	**0.84**	**0.48**	**0.5**
400	0.68	0.94	0.91	0.83	0.48	0.5

Comparison of prediction results between SVM-RFE-CBR dimension reduction and original dimension. The bold values mean maximum value in the column.

The experimental results show that both Acc and MCC are improved after feature dimensionality reduction, which eliminates the redundant part of the original feature and improves the performance of the model.

### 3.4 Comparison of Different Classifier Performance on Dataset

With the development of computers, machine learning has been widely used in bioinformatics (Tang et al., 2019; Wang et al., 2020c; Fu et al., 2020; Cai et al., 2021; Wang et al., 2021c; Jin et al., 2021), and there are many classification models, including the linear classifier, SVM, naive byes, K-nearest neighbor (KNN), decision tree (DT), and ensemble model (random forest/GDBT, etc.). To obtain the most effective classifier method to identify SNARE proteins, we use various machine learning classifiers to construct a model of SNARE protein recognition, including random forest, KNN and naive Bayes.

We compare the experimental results of multiple machine learning classifier training models with the performance measurement results. The performance result of different classifier shown in Table 3.

**TABLE 3:** The result of performance compares between SVM and other classification method.

	Sn	Sp	Acc	MCC
KNN	**0.870**	0.906	0.898	**0.73**
Random Forest	0.620	0.962	0.900	0.70
Naïve Bayes	0.853	0.595	0.624	0.28
SVM	0.650	**0.970**	**0.900**	0.70

The result of performances compares between SVM and other classification method. The bold values mean maximum value in the column.

As we can observe from Table 3, the results of SVM on training dataset are better than another classifier.

**FIGURE 3:**
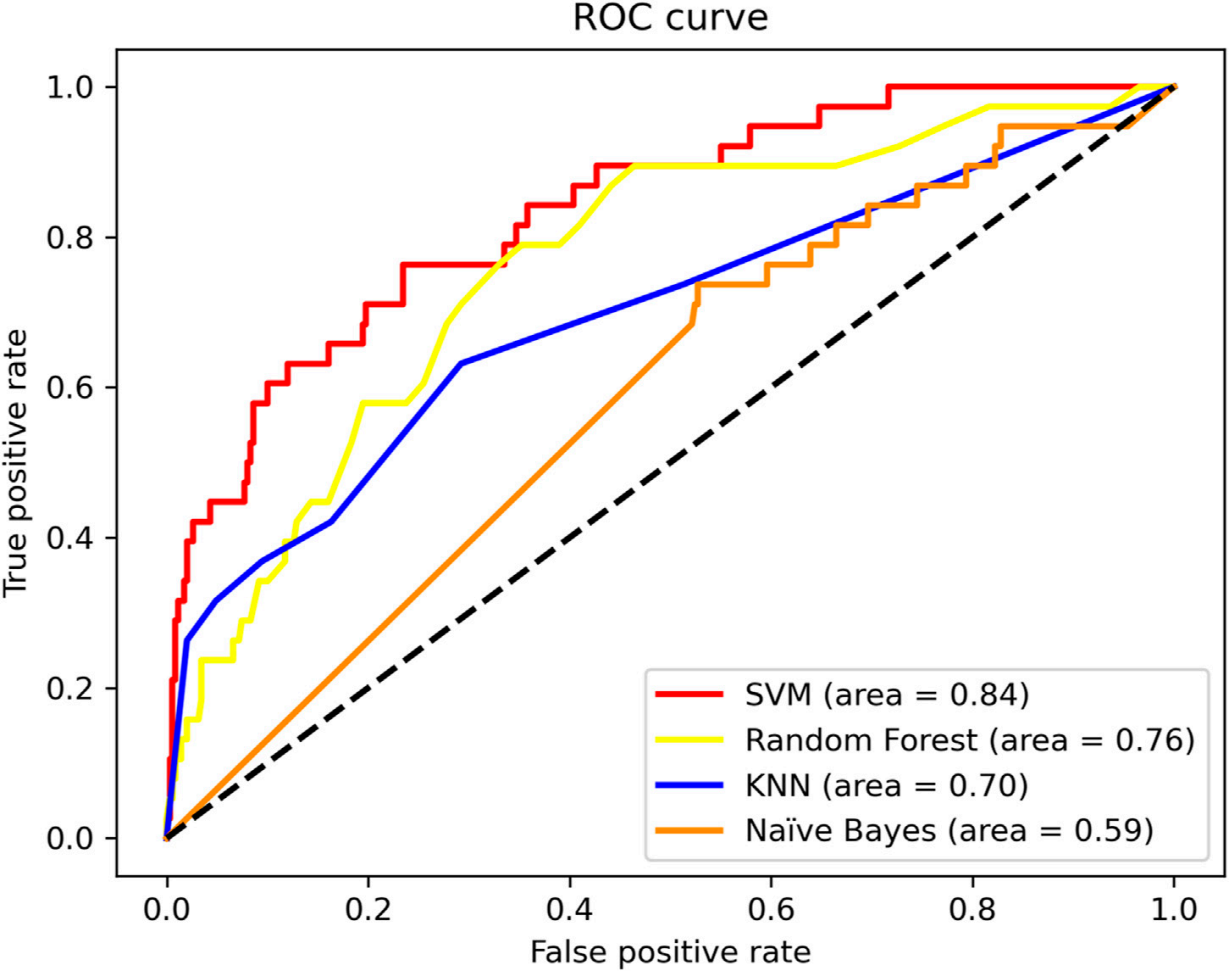
ROC curves of different classifier methods.

In particular, Sp = 0.970, Acc = 0.900. SVM shows higher performance. Meanwhile, we compare the ROC curves of different classifier method. The result shown in Figure 3. As we can observe from Figure 3, The ROC curve of SVM is obviously better than the other three classifiers.

### 3.5 Comparison of Different SNARE Protein Identification Methods

**FIGURE 4:**
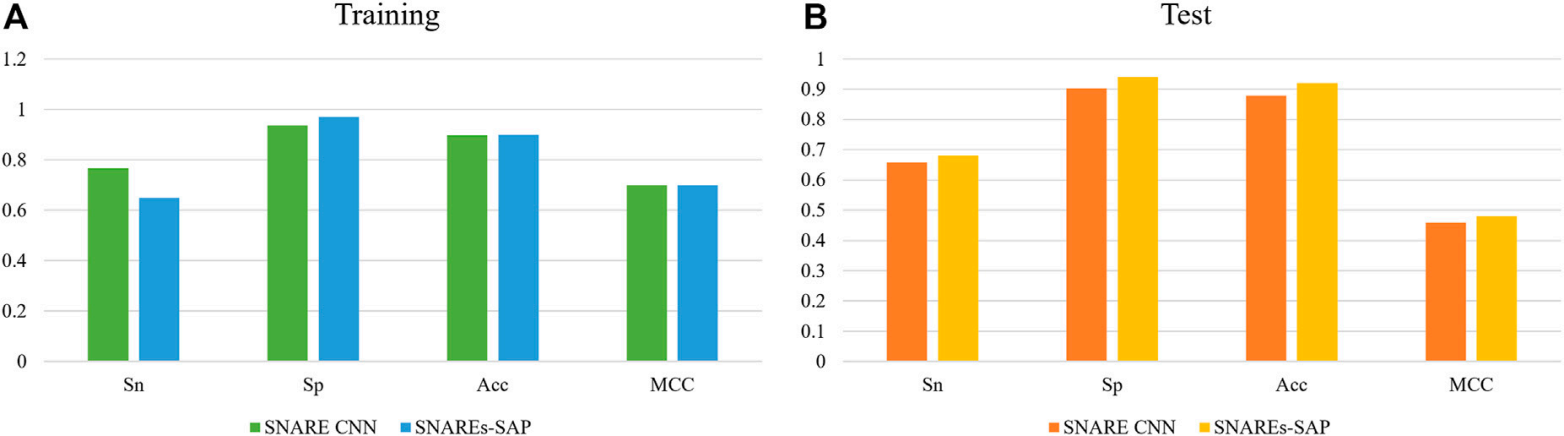
**(A)**The result of performance compares between our classification method and other classification method on training datasets **(B)** The result of performance compares between our classification method and other classification method on test datasets.

We compare the experimental results of SNARE CNN with the performance measurement results of our research method. The independent test results of using different methods to identify SNARE proteins are shown in Figure 4. Figure 4A shows the result of performance compares between our classification method and other classification method on training datasets. Figure 4B shows the result of performance compares between our classification method and other classification method on test datasets.

The results show that our method gives good results in both training and independent test datasets. To compare the performance measurements of our method for identifying SNARE proteins with other methods more accurately, we compare the results of different methods on independent test datasets.As we can observe from Figure 4B, the independent test results of our method are better than SANRE CNN. Sn = 0.68, Sp = 0.940, Acc = 0.92 and MCC = 0.48, and all these indicators reach the highest values using our method. As shown above, our method shows higher performance. These results clearly demonstrate the superiority of our method over the existing methods, especially when using an independent dataset test. This means that our method can better recognize SNARE proteins.

## 4 Discussion

Because of the importance of SNARE proteins and the vital significance of SNARE proteins in vesicular transport, there is an urgent need for classification methods to identify SNARE proteins. Extracting meaningful features and selecting an appropriate machine learning algorithm can greatly increase the model performance of protein prediction. We propose a method based on PSSM profiles to extract features and SVM to construct a model to identify SNARE proteins. We normalize the feature data and use the SVM-RFE-CBR algorithm to reduce the dimensions of feature. Then, we use a 10-fold crossing validation training model and use an independent dataset to test the performance of the model (Li et al., 2017; Li et al., 2020c). The accuracy, specificity, sensitivity, AUC, MCC and other performance indicators of our method have excellent experimental results. All results show that our model has better performance than other machine learning methods and advanced neural networks. Our method can effectively identify SNARE proteins. Taken together, the method proposed in our study is of great significance for the study of SNARE proteins and may also contribute to the prediction of protein function. Future works may include investigation of more kinds of proteins.

## Data Availability

The datasets presented in this study can be found in online repositories: https://github.com/First-Leaner/Identify-proteins. The names of the repository/repositories and accession number(s) can also be found in the article/Supplementary Material.
